# Economic and value chain analysis to support an investigation and risk mitigation efforts on Marek’s disease in layers in the southern part of Thailand

**DOI:** 10.14202/vetworld.2023.35-45

**Published:** 2023-01-09

**Authors:** Tosapol Dejyong, Karoon Chanachai, Tippawon Prarakamawongsa, Wandee Kongkaew, Anyarat Thiptara, Taweesak Songserm, Theera Rukkwamsuk, Damian TagoPacheco, Waraphon Phimpraphai

**Affiliations:** 1 Food and Agriculture Organization of the United Nations, Regional Office for Asia and the Pacific, Phranakorn, Bangkok, Thailand, 10200; 2 Graduate Student, Bio-Veterinary Science Program (International), Faculty of Veterinary Medicine, Kasetsart University, Bangkok, Thailand, 10900; 3 United States Agency for International Development, Regional Development Mission Asia, Patumwan, Bangkok, Thailand, 10330; 4 Regional Field Epidemiology Training Program for Veterinarians, Department of Livestock Development, Ratchathewi, Bangkok, Thailand, 104004; 5 Veterinary Research and Development Center (Upper Southern Region), National Institute of Animal Health, Department of Livestock Development, Thung Song, Nakhon Si Thammarat, Thailand, 80110; 6 Department of Veterinary Pathology, Faculty of Veterinary Medicine, Kasetsart University, Kamphaeng Saen, Nakhon Pathom, Thailand, 73140; 7 Department of Large Animal and Wildlife Clinical Science, Faculty of Veterinary Medicine, Kasetsart University, Kamphaeng Saen, Nakhon Pathom, Thailand, 73140; 8 Department of Veterinary Public Health, Faculty of Veterinary Medicine, Kasetsart University, Kamphaeng Saen, Nakhon Pathom, Thailand, 73140

**Keywords:** birds, case–control study, risk factors, Thailand

## Abstract

**Background and Aim:**

Marek’s disease (MD) is a common lymphoproliferative disease affecting chickens and causing economic losses in commercial poultry. The MD outbreak was noticed in the southern part of Thailand in 2019. The suspected cases were found with an abnormal number of cases of layers dying with clinical signs, for example, weakness and emaciation, with evidence of MD gross lesions. This study aimed to raise awareness of the MD outbreak through value chain analysis (VCA), identifying associated possible risk factors, and estimating the associated economic impact.

**Materials and Methods:**

Value chain analysis, including seasonal calendar, value chain diagram, and layer movement mapping of the layer industry, was conducted. High-risk stakeholders were identified on the basis of risk practices and interactions between stakeholders. A case–control study was conducted to determine risk factors associated with the MD outbreak on layer farms, and partial budget analysis was used to estimate economic losses associated with MD.

**Results:**

The value chain diagram showed the linkages between stakeholders, including estimation of the percentage of products moved from one stakeholder group to another and the negotiated price. Fourteen out of 35 layer farms were case farms. Farm size and source of birds were significantly associated with the MD outbreak. The MD outbreak caused total economic losses of 295,823 USD. Farms that slaughtered infected birds with additional revenues incurred losses of 140,930 USD, whereas farms that culled infected birds without additional revenue returned incurred losses of 1995 USD.

**Conclusion:**

The VCA provided a better understanding of the layer and egg businesses in South Thailand and guided the development of questionnaires for outbreak investigation. The potential risk factor findings suggested the need for further exploration of the source of the MD outbreak.

## Introduction

Marek’s disease (MD) is an immunosuppressive disease and a contagious lymphoproliferative disorder in poultry, including quail, turkeys, ducks, and chickens. Marek’s disease is not a zoonotic disease; however, it can have a big impact on the poultry industry. Chickens are more susceptible to the disease than other livestock as they are an important reservoir host for the MD virus (MDV) [1].

Marek’s disease is caused by an alpha-herpesvirus, which can induce lymphoproliferation in the infected poultry. The disease can cause lymphomas in visceral organs and tissues, and enlargement of the peripheral nerves due to lymphocytic infiltration [2, 3]. Three serotypes of MDV have been identified: Serotype 1 (MDV-1) (a high virulent strain), serotype 2 (MDV-2) (an avirulent strain used as a vaccine), and serotype 3 (MDV-3) (herpesvirus of turkeys, used as a vaccine) [1, 4]. Clinical signs of MD include depression, reduced growth rates, inactivity, anorexia, and decreased egg production. Due to multiple lymphoid tumors in visceral organs and neurological degeneration, MD-specific clinical signs such as neurologic disorders, paralysis (lameness), and blindness can also appear [1, 5–8]. Marek’s disease outbreaks cause variable prevalences of ocular or “gray eye,” for example, >90% of infected birds in the USA in 1990 showed blindness [9], whereas only one out of 28 positive chickens in backyard chickens in California, USA, showed ocular signs [10].

Clinical signs are seen between 8 weeks and 17 weeks of age [11]. Morbidity and mortality rates ranging from 2.01% to 9.15% and 1.03% to 7.6%, respectively, have been recorded in commercial layer flocks in India [4]. In Thailand, morbidity and mortality rates in commercial layer flocks were estimated at 9.59% (range: 1.72%–73.34%) and 4.82% (range: 0.04%–66.67%), respectively [12].

Widespread vaccination against MD was introduced in Thailand during the 1970s. Since then, MD morbidity and mortality have drastically reduced; however, sporadic MD outbreaks are reported globally and vaccine breaks do occur due to the evolution of more virulent MDV pathotypes [4, 8].

The MDV matures into a fully infective particle in the feather follicle of MDV-infected birds. Newly introduced unvaccinated birds, especially young chicks, which are highly susceptible, can be infected if they come into contact with an infected flock [11]. Airborne transmission is the main mode of transmission of MDV as a virus particle in the feather follicle can survive for a month in litter or dust. This is an important factor in the transmission of MDV from the dust due to poor sanitation of poultry houses, inadequate downtime period, overstocking or high density, immunosuppression, stress and coinfection with other diseases, lack of vaccination, and contamination through fomites (hands, clothing, shoes, hair, and skin). Infected birds can spread the disease throughout their whole life [4, 7, 11].

The economic burden of MD is attributed to additional morbidity and mortality in broilers or laying birds, degradation of the bird’s value, egg production loss, culling of diseased birds, and costs of additional disease prevention and control measures, including vaccines [13, 14].

Value chain analysis (VCA) can support disease outbreak investigation, especially for animal diseases such MD, which has a strong association with the animal movement. For instance, in Europe, the VCA approach was used for backward and forward tracing in food-borne disease outbreak investigations [15]. Data collected from VCA can be used for epidemiological risk analysis and other risk-based approaches for disease outbreak investigation. Risk pathways can be generated using value chain diagrams and information on the disease situation. Biosecurity and management practices can be assessed to identify risk levels and critical control points, which can be used as baseline information for risk-based planning to guide outbreak investigation and risk mitigation efforts [16]. Data can also be used to identify areas where the disease could be present so that timely control measures are implemented to minimize the impact of the outbreaks. Therefore, conducting VCA before an outbreak happens is recommended as it will facilitate and expedite the investigation when the disease is detected.

Although, MD vaccine is used in some farms in Thailand, awareness of the need for disease notification and reporting are low among farmers in Thailand [17, 18]. Although the disease now rarely occurs, it can cause high and persistent losses for the egg industry. In 2019, the Upper-Southern Veterinary Research and Development Center (VRDC) noticed an abnormal number of cases of layers dying from weakness and emaciation, with evidence of gross lesions in some cases, which could be related to MD. When samples were collected and tested, the presence of the MDV was confirmed.

This study aimed to raise awareness of the MD outbreak by investigating the value chain of the layer and egg industry in the Nakorn Sri Thammarat Province and estimating the economic impact associated with the MD outbreak. The results were used to better understand the outbreak situation and identify possible risk factors associated with the outbreak.

## Materials and Methods

### Ethical approval and Informed consent

This study was jointly developed under the authorization of Nakhon Si Thammarat Provincial Livestock Office and under the Regional Field Epidemiology Training Program for Veterinarians, Department of Livestock Development, Thailand. According to the nature of the study of outbreak investigation and the low risk posed to the participants, formal approval from an ethics committee was not a requirement. No samples from animals were collected under this field study; the samples were collected from diseased chickens during outbreaks before the study. Questionnaires and existing data of the government were the main sources of data that were used for the analysis.

The response of all stakeholders for the VCA and the farmers was based on their knowledge of normal practice, management, and economic information of their farms. Moreover, before organizing the value chain workshop and questionnaire interview, the team of researchers, including local officers, explained the aims of the study to all stakeholders, and after the analysis was done, the researchers provided results and recommendations back to them. Any confidential issues of individual interviewees were kept secret.

### Study period and location

The study was conducted during November and December 2019 in the southern part of Thailand in collaboration with the Veterinary Research and Development Center (Upper Southern Region), Nakhon Si Thammarat Provincial Livestock Office, and the Regional Field Epidemiology Training Program for Veterinarians (R-FETPV), Department of Livestock Development (DLD).

### Study design and data collection

#### Value chain analysis

A cross-sectional study was conducted on the layer industry in the Nakorn Sri Thammarat Province, Thailand. Six stakeholder groups in the layer industry, including layer farms, pullets, and day-old chicks (DOCs) suppliers with and without parent stock farms, slaughterhouses, an egg collecting center, and local veterinary officers, were interviewed. Convenience sampling and quota sampling with representatives of each stakeholder group were used and the number of interviews contacted depended on time and financial constraints as well as the availability of the stakeholder. The participants in the study included the following: Seven out of 40 affected layer farmers, one representative of the pullet and DOCs supplier-with-parent-stock farms, three representatives of supplier-without-parent-stock farms, one representative out of five registered slaughterhouses, one representative out of three egg collecting centers, and four local veterinary officers.

Inputs from stakeholders were obtained during a workshop using semi-structured interviews and participatory epidemiological methods. The interview consisted of three sessions:

- Session 1 – Seasonal calendar development: Percentage of production of eggs, spent hens, DOCs, and pullets were estimated by month, to capture the seasonality of movements
- Session 2 – Value chain diagram development: Stakeholders associated with layer chickens and the egg trade as well as their linkages, were identified. The proportion of products moved through each channel was evaluated and market prices were collected
- Session 3 – Risk practices identification: Stakeholders were interviewed using guiding questions to assess risky practices associated with MD. A layer movement map was developed using the animal movement database of pullets and DOCs in 2019 from the Department of Livestock Development, Thailand. The movement map was developed using all movements of layer chickens originating from different parts of the country destined for the Nakorn Sri Thammarat Province.

#### Outbreak investigation and estimation of economic losses

**Figure-1:**
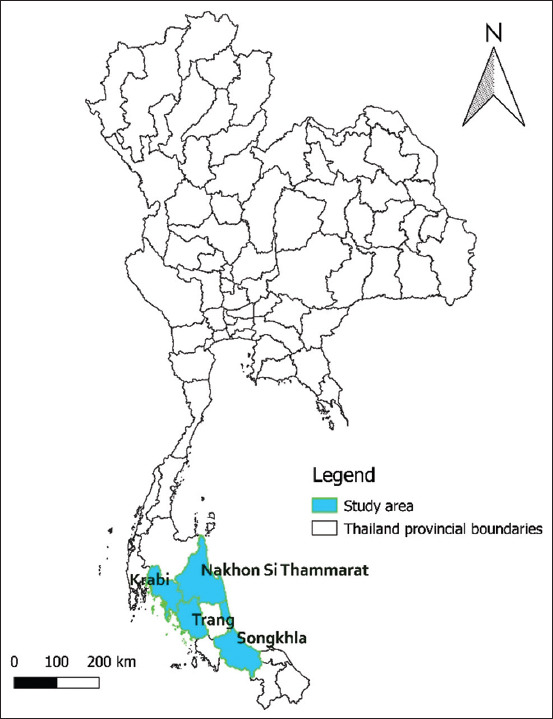
Study area of outbreak investigation and estimation of economic losses. The study was conducted using information from Nakorn Sri Thammarat, Trang, Songkhla, and Krabi, which are labeled as yellow areas [Source: QGIS software version 3.4].

A case–control study was conducted to identify risk factors associated with the MD outbreak on layer farms in the southern part of Thailand in 2019. Almost all layer farms in the Nakorn Sri Thammarat Province (87.5%) and layer farms in other provinces in the southern part of Thailand, such as Trang, Songkhla, and Krabi (12.5%) that notified of MD were included in the study (Figure-1).

A suspected case of MD was defined as (1) a layer farm in southern Thailand with a higher mortality rate in 2019 than in 2018 with an unknown cause or (2) layers in at least one house on the farm with at least four of the following clinical signs since January 2019: Growth retardation or non-uniformity at the same age, stunted growth, emaciation, depression, transient paralysis of legs or wings, ataxia, or reduced egg production at peak (<90%–95%).

Laboratory confirmation of MD was conducted using gross and histopathological diagnosis together with conventional polymerase chain reaction (PCR) techniques for detecting the *meq* gene on MDV. The presence of a *meq* gene indicated that the isolate had an oncogenic gene and was pathogenic [19–21].

For outbreak investigation, a farm was considered as a case farm for MD if a farm met the suspected case definition and was confirmed by PCR or histopathology since January 2019. A non-case farm was a farm that did not meet the suspected case definition or was confirmed negative for MD by PCR or histopathology since January 2019 by the VRDC.

Results from VCA, especially, captured risk practices, and production systems were used to develop questionnaires. The main objective of the questionnaires was to guide interviews and collect data on general farm information and animal demographics, clinical signs and abnormal events, farm management and risk factors, and economic losses due to the disease.

### Statistical analysis

#### Value chain analysis

The data collected through the workshop were entered into the Excel spreadsheet for qualitative and quantitative analyses. The seasonal calendar, value chain diagram, and layer movement mapping were developed. High-risk stakeholders were identified based on risk practices and interaction between stakeholders using the definitions outlined (Table-1) [22].

**Table-1:** Qualitative likelihood scale categories for identifying high-risk stakeholders [22].

Category	Definition
High	There is more than an even chance that the event will occur
Moderate/medium	The event could occur, but it is unlikely
Low	The event is very unlikely to occur

### Outbreak investigation

Data gathered during the interviews were entered and managed by an Excel spreadsheet for verification and validation. Descriptive statistics of case farms were computed according to potential risk factors for MD, including farm characteristics and farm management practices. To identify the strength of the association between potential risk factors and MD, univariate and multivariate analyses were conducted to calculate the odds ratio with a 95% confidence level. Pearson’s Chi-squared tests (or Fisher’s exact tests) were conducted for the univariate analysis and logistic regression (using Epi Info version 7.2.2.6) was used for the multivariate analysis.

#### Estimation of economic losses of MD

Economic losses associated with MD were calculated using partial budget analysis. Total losses were calculated from additional costs and reduced revenues. These included loss due to medical and laboratory costs, loss due to egg reduction because of MD infection, loss due to culled, slaughtered or dead birds (as some farms preferred to cull their birds themselves without money returned whereas some farms sent birds to slaughterhouse for money), and production loss due to the interrupted cycle. On the other hand, total savings were calculated from reduced costs, which include reduced labor and feed costs, and additional revenues. For additional revenues, we considered two scenarios: (1) Farms with additional revenues due to compensation from insurance and slaughtered suspected hens and (2) farms without additional revenue as infected birds were culled with no money in return (Table-2).

**Table-2:** Economic losses of Marek’s outbreak at the farm level using partial budget analysis.

Problem: Marek’s outbreak at the farm level			

Additional costs		Additional revenues	
– Loss due to medical and laboratory costs	Cost of medicine + cost of disinfectants + cost of laboratory + cost of veterinarian	– *Increased income due to compensation and slaughtered suspected hen	Compensation + (number of slaughtered birds*spent hen price)
Reduced revenues		Reduced costs	
– Loss due to egg reduction	Average number of egg reduction per day*number of days of reduction*egg price	– Reduced labor cost	Salary of workers per hour*work-hour-saved
– Loss due to culled, slaughtered, and dead birds	Number of culled and slaughtered birds*spent hen price	– Reduced feed cost	Number of culled, slaughtered and dead birds*feed cost per layer per day*(Normal age at spent hen – age (day) at culling)
– Production loss due to interrupted cycle	Number of culled, slaughtered and dead birds*(Normal age at spent hen – age at culling) *egg price*number of eggs per day		
Total losses=Additional costs+Reduced revenues		Total savings=Additional revenues+Reduced costs	

Net change in profit (economic losses) = Total losses - Total savings

Note: Relevant for the first scenario: Case farms with additional revenues due to compensation from insurance and slaughtered suspected hen.

Assumptions:

1. According to the interviews, the average age at which layers are sold as spent hens is 532 days (76 weeks)
2. The costs of laboratory and veterinary service to diagnose and control the disease are zero since we are estimating the impact of the outbreak from the farms’ perspective and the diagnostics is provided by the government free of charge. Furthermore, the operating costs for culling and disposing of birds (burying or burning) are zero, according to the interview.

## Results

### Value chain analysis

**Figure-2:**
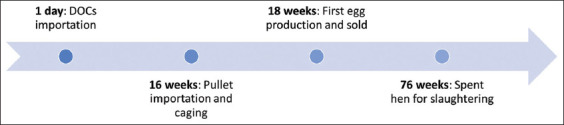
Pattern of layer industry in Nakhon Si Thammarat province.

As a common practice, layer farms in the area introduce replacement layers by purchasing both DOCs and pullets at <16 weeks old. At 16 weeks, pullets are moved to cages and start producing eggs sold to markets at 18 weeks. Layers produce eggs until they are 76 weeks old on average, after which they become spent hens due to their reduced egg production, and are marketed for local consumption (Figure-2).

**Figure-3:**
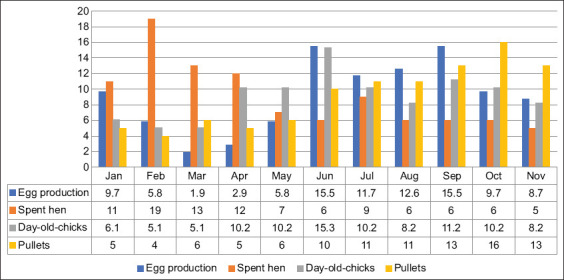
Seasonal calendar of relevant layer productions of Nakhon Si Thammarat province. Four types of commodities are represented in different lines. Percentages of 11 months of each commodity add up to 100%.

According to the interview and agreement from the representative stakeholders in the workshop, production of DOCs and eggs peaks in June, whereas production of spent hen peaks in February. The introduction of pullets peaks in October (Figure-3).

**Figure-4:**
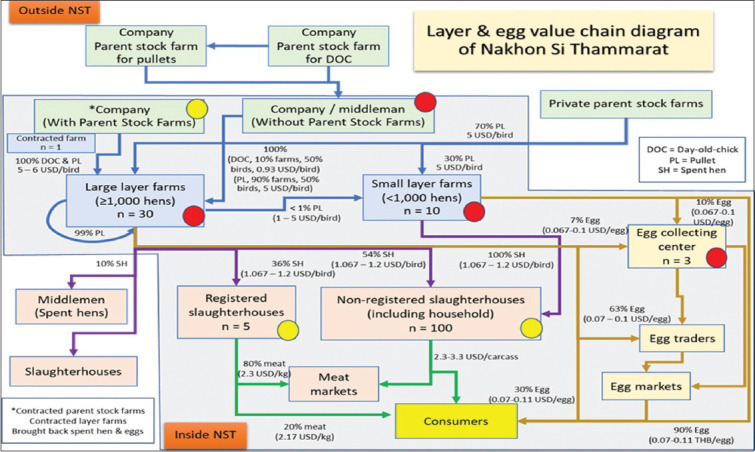
Value chain diagram of layer *industry of Nakhon Si Thammarat province*. In this scheme, the gray area represents the territory within Nakhon Si Thammarat province, while the white area represents the area outside the province. The diagram consists of value chains of four commodities: The value chain diagram of day-old chicks and pullets is represented by the blue line, the value chain diagram of spent hen is represented by the purple line, the value chain diagram of egg is represented by the brown line, and the value chain diagram of chicken meat is represented by the green line. The numbers of each stakeholder are identified under stakeholder boxes (n). For each interaction or arrow, the volume traded is estimated as a percentage (%) and prices per unit in USD.

Stakeholders of the layer and egg industry in the area were identified based on consultation with the Southern Layer Farmer Association. The value chain diagram (Figure-4) developed by stakeholders shows the linkages between stakeholders of the layer industry in the province (within the gray area), including an estimation of the percentage of products moved from one stakeholder group to another and the price negotiated. Risky practices along the value chain were identified and used to support the development of questionnaires for the MD outbreak investigation. The top three risky practices were as follows: (1) A company without parent stock farms and did not have information about the MD vaccination program as the chickens are normally obtained from other companies/farms and when there is a high demand, new chicken supplier companies will be engaged to increase chicken supply in the area, (2) use of the same vehicles for spent hen transportation to slaughterhouses for many farms without appropriate cleaning or disinfection, and (3) at egg collecting centers, egg trays were normally rotated and shared among farms without appropriate cleaning or disinfection, vehicles for egg transportation were used for many farms without appropriate cleaning or disinfection, and egg sizing machines were regularly cleaned but without disinfection. Based on such risky practices, risky stakeholder groups were identified. High-risk stakeholders (red dot) were a company or middleman without parent stock farms, both large- and small-scale layer farms, and egg collecting centers. Moderate-risk stakeholders (yellow dot) were a company or middleman with parent stock farms, and both registered and non-registered slaughterhouses.

**Figure-5:**
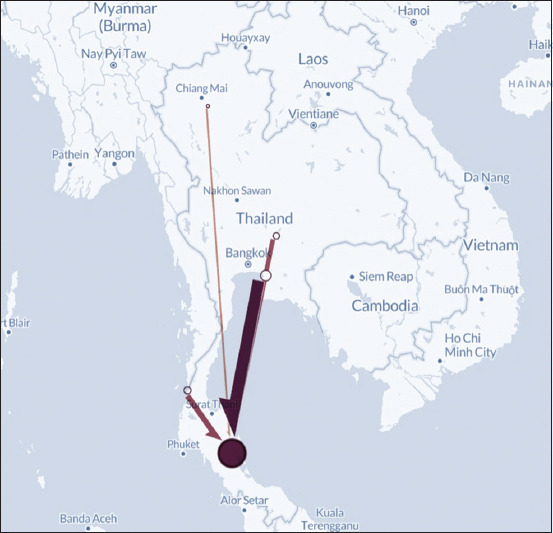
Layer movement mapping of day-old chicks and pullets to *Nakhon Si Thammarat, Trang, Songkhla*, and *Krabi* provinces. The intensity and lightness of each line represented frequency of movements [Source: Official information from the DLD and produced by FlowmapBlue. https://flowmap.blue/].

From the animal movement database, we extracted the movements of DOCs and pullets in the egg industry in the studied provinces. These movements originated from different parts of Thailand but the major source of DOCs and pullets was East Thailand, from where the companies without parent stock farms brought their DOCs and pullets (Figure-5).

### Descriptive analysis from outbreak investigation

#### Farm characteristics and management practices

A total of 35 layer farms were interviewed. Most of the farms were small (<10,000 layers each) (n = 22/35, 63%), with closed housing or with net installed (n = 24/35, 69%), raising native chickens (n = 26/35, 74%), without MD vaccination certificates for the birds purchased from other provinces (n = 6/9, 67%), and with a median experience of 20 years. The majority of farms used battery or frame cages (65%) and the average density of birds per cage was 0.04 heads/m^2^ (four birds in 0.16 m^2^). Almost a third of the farms recorded a higher mortality in 2019 than in 2018 (n = 11/35, 31%).

There were four sources of DOCs or pullets:

1. 20% of farms purchased birds from Company A,
2. 43% of farms purchased birds from Company B,
3. 23% of farms purchased birds from Company C, and
4. 23% of farms purchased birds from other unidentified sources.

In addition, 35% of farms changed feed supply companies in 2019, and 56% of farms had no barrier to prevent animals from outside layer houses. Similarly, 60% of farms showed that vehicles could enter their farms without proper cleaning, and 60%–86% of farms stated that their workers did not change their clothes, shower, or dip their booths before entering the farms. Approximately 56% of farms reported that their workers worked across layer houses and 38% of them shared farm equipment between houses.

Moreover, 61% of the farms did not quarantine before introducing new birds, with a few of them mixing birds of different ages (23%) and from different sources (14%) in the same house. The median length of the downtime period of the farms was 60 days ranging from 14 to 120 days. Regarding egg tray management, 33% of farms mixed inside egg trays and outside egg trays and ~70% of farms did not clean outside egg trays with disinfectant daily. Most of the farms (73%) did not clean feed and water equipment with disinfectant. Whereas 83% of the farms reported vaccinating their birds against Newcastle, no farm vaccinated its birds against MD.

**Figure-6:**
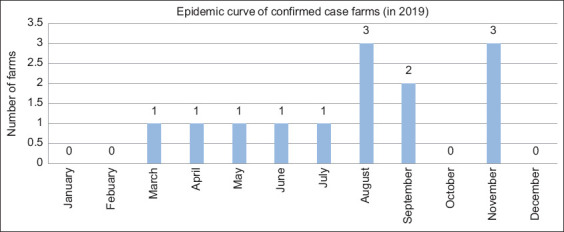
Epidemic curve of confirmed case farms.

The VRDC confirmed 14 farms out of 35 as case farms. The highest number of layer case farms (11/14) was in the Nakhon Si Thammarat Province, whereas Trang, Songkhla, and Krabi Provinces registered just one case each. Using data from 13 case farms (onset date of one case farm was not clearly reported), epidemic curve of the MD was produced (Figure-6). The disease was first reported in March 2019, with peaks in recorded outbreaks in August and November 2019.

Investigations in 11 of the case farms showed that the median age of sick birds was 31 weeks. Morbidity and mortality data were available for only five and eight case farms, respectively. Morbidity averaged 10% and mortality averaged 8%. The case fatality rate was computed for five case farms and averaged 75%. Only four out of seven case farms reported that they disposed of sick/dead birds by burning or burying.

**Figure-7:**
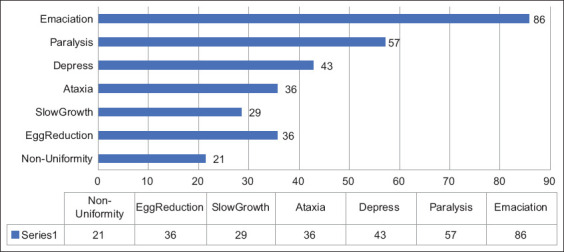
Clinical signs of case farms.

Emaciation was the major clinical sign of birds infected with MD (85.7%), followed by paralysis (57.1%), depression (42.9%), ataxia (35.7%), egg reduction (35.7%), slow growth (28.6%), and non-uniformity in a flock (21.4%) (Figure-7).

#### Inferential analysis from outbreak investigation

Out of 34 factors, 14 factors that were commonly reported by case farms but not by control farms were selected for the univariate analysis. Two factors (farm size and source of birds) were subjected to further analysis as they were significantly associated with the MD outbreak (p > 0.1) (Table-3). In the final multivariable model, no variable was identified as significantly associated with the MD outbreak (Table-4).

**Table-3:** Univariate analysis with 14 variables.

Variables	Odds ratio	Risk ratio	p-values
Farm size (>10,000 heads)*	4.07 (0.95–19.29)	2.26 (1.01–5.05)	0.049
1. Bird supply from Company A*	5.01 (0.66–62.3)	2.2 (1.09–4.5)	0.07
2. Bird supply from others	3.21 (0.5–25.6)	1.88 (0.88–4)	0.22
3. Battery cage or A frame	0.41 (0.08–2.07)	0.55 (0.2–1.5)	0.25
4. Source of sick birds from Company A	-	1.5 (0.95–2.4)	0.5
5. Feed company changed in 2019	2.09 (0.48–9.5)	1.57 (0.68–2.6)	0.3
6. Workers do not change cloth before entering farms	2.34 (0.33–27.9)	1.78 (0.5–6.3)	0.4
6. Workers do not dip boots before entering farms	2.22 (0.45–13)	1.66 (0.65–4.3)	0.31
7. No poultry quarantine before introducing a farm	1.77 (0.33–11)	1.42 (0.56–3.6)	0.48
8. Mix egg tray (inside and outside)	2.66 (0.59–12.7)	1.79 (0.79–4.07)	0.18
9. Not clean outside egg tray with disinfectant	0.37 (0.07–1.93)	0.53 (0.2–1.4)	0.21
10. Not daily clean egg tray	0.41 (0.06–3)	0.57 (0.22–1.5)	0.39
11. Not clean feed and water equipment with disinfectant	2.44 (0.35–28.9)	1.88 (0.5–6.95)	0.43
12. Equipment shared between house	0.5 (0.08–2.54)	0.65 (0.26–1.63)	0.47

* *p* < 0.05 is considered as a statistically significant risk factor

**Table-4:** Multivariate analysis.

Variables	Estimated coefficient	p-values
1. Farm size (>10,000 heads)	−0.00003188	0.21
2. Bird supply from Company A	13.94	0.15

#### Estimation of economic losses of MD in 2019

Only nine case farms provided enough economic data to estimate economic losses, which amounted to 295,823 USD. Total economic loss was calculated based on losses due to egg reduction (21,559 USD), loss due to culled, slaughtered and dead birds (16,565 USD), production loss due to the interrupted cycle (411,361 USD), loss due to medical and laboratory costs (2620 USD), and additional revenues (increased income due to compensation and slaughtering of suspected hens (26,220 USD) and reduced costs (labor cost, 1033 USD and feed costs, 129,029 USD) (Table-5). Apart from the total losses, there were two farms that slaughtered infected birds with additional revenues due to compensation from insurance; the average economic losses on these farms were 140,930 USD. Another seven farms culled the infected birds without additional revenue returned as they had no insurance; the average losses from these farms were 1995 USD. However, five farms provided no information on their economic losses.

**Table-5:** Estimation of economic losses of Marek’s disease in 2019 for case farms (n=9).

Problem: Marek’s outbreak at farm level			

**Additional costs**		**Additional revenues**	
– Loss due to medical and laboratory costs	2620 USD	– *Increased income due to compensation and slaughtered suspected hen	26,220 USD
Reduced revenues:		Reduced costs:	
– Loss due to egg reduction	21,559 USD	– Reduced labor cost	1033 USD
– Loss due to culled, slaughtered and dead birds	16,565 USD	– Reduced feed cost	129,029 USD
– Production loss due to interrupted cycle	411,361 USD
Total losses=452,105 USD		Total savings=156,282 USD	
Net change in profit (economic losses) = 295,823 USD			
Average loss of all case farms (nine farms) = 32,869 USD			
Average loss of case farms under the first scenario (two farms) = 140,930 USD			
Average loss of case farms under the second scenario (seven farms) = 1995 USD			

* Relevant for the first scenario: Case farms with additional revenues due to increased income for which the farms received compensation from insurance and slaughtering suspected hens.

## Discussion

Although MD is a notifiable disease in Thailand, it is also a neglected disease. Field epidemiological investigations on MD are not a common practice in Thailand. No proper investigations have been recorded over 20 years. Moreover, the layer and egg industry of Thailand mostly consists of small commercial farms, which have inaccurate information on production and veterinary records, making quantitative analysis even more challenging. Marek’s disease outbreaks have been investigated in other countries, including Ethiopia [23] and India [4]. Only a few studies of disease are available, especially, epidemiological studies and outbreak investigations.

The value chain diagram is useful to investigators for tracing backward and forward. In studies with limited information, VCA helps in capturing all stakeholders in the value chain as well as their interactions in complex markets and in examining the inter-relationships between diverse actors involved in a particular area, including interactions between commercial and smallholder systems [24, 25].

When building the seasonality calendar, the interviewees stated that the egg price in 2019 was very high. This incentivized farmers to increase the number of DOCs and ready-to-lay pullets to maximize their egg production and take advantage of the high egg price. To meet their demand, farmers used new sources of DOCs and pullets. Similarly, a study of egg price stabilization in Thailand showed that the balance of amount of egg production, price, and consumer demand would substantially influence management of layer production [26].

Farms did not conduct vaccination against MD, and opportunistic bird suppliers grabbed the opportunity to benefit from the high demand for replacement stock. New chicken suppliers without knowledge of MD and MD vaccines were engaged in increasing chicken supply. Marek’s disease vaccination, however, can cause the evolution of higher virulence strains in the field [8, 27]. The value chain diagram indicated that small-scale farms had no power to negotiate the price of DOCs due to their small purchases, forcing them to buy some DOCs from large farms. Thus, large farms, especially those that ordered DOCs and pullets with risk of MDV infection from other areas, were identified as critical stakeholders to be included in the outbreak investigation. The critical role of large farms was confirmed by the outbreak notification report from the VRDC, which stated that large farms were the main MD case farms.

The value chain approach can also be used to increase awareness of the disease among key stakeholders and understanding factors that contribute to disease introduction and spread among stakeholders. The interviews of stakeholders helped to identify risky practices and challenges associated with the poultry value chain, such as lack of sufficient low-risk bird suppliers. Some of the risky practices identified included sharing vehicles between farmers for transporting poultry to slaughterhouses, rotating egg trays among farms without appropriate cleaning or disinfection, and sharing vehicles for egg transportation. These risky practices were identified through the VCA and awareness was raised among stakeholders identified as risky nodes in the value chain.

Because of the chronic form of the disease, the onset of the development of tumors is as early as 14 days after infection [28], and its incubation period can be 1–4 months [29]. These characteristics can explain why the number of outbreaks accelerated in August, September, and November 2019, which corresponded to a few months after the season of increased demand and transportation of DOCs (peak in June) and pullets (peak in October) to the area. From the previous studies, the mortality rate of MD is around 10%–15% [9, 21], which is similar to the mortality rate of 10% recorded in this study.

Clinical signs of infected birds are most commonly seen between 12 and 30 weeks of age [30], and MD outbreaks usually occur from 32 to 47 weeks of the production cycle [31], which matches the median age of sick birds of 31 weeks recorded in this study. The major clinical manifestation observed in this study was depression (43%). Most infected birds exhibited the chronic or classical form with the following clinical signs: Emaciation (85.7%), paralysis (57.1%), ataxia (36%), egg reduction (36%), slow growth (29%), and non-uniformity (21%). These findings were slightly different from those of the study of MD in chicken strains in Ethiopia, in which the majority of infected chickens were infected with the acute form [32]. The gray eye form was not found in this study, although it was included in the questionnaire, but it was difficult for farmers to detect this clinical form.

Immunization against MD in DOCs plays a crucial role in the epidemiology of MDV transmission, as Marek’s vaccines have high efficacy [21, 33]. From the univariate analysis of the potential risk factors, a large farm size (>10,000 birds) was associated with the disease outbreak (p < 0.1). This may be attributed to the increased demand for DOCs and pullets, which led to buying birds from unknown suppliers without following the appropriate quality assurance procedures. One of those new suppliers, Company A, was associated with the disease (p < 0.1). When conducting the investigation, Company A started as a feed company, but to meet the high demand of farmers during that period, the company turned into a supplier of DOCs and pullets. Therefore, the MD disease vaccination program should be further promoted. Although, farm management and hygiene factors were not significantly associated with the disease, those factors must be considered important as they associate with increased mortality and magnitude of the outbreak, virus introduction, and shedding of the disease in the area [33].

The economic losses at the farm level were not homogenous. Some farms that intended to eliminate the disease from their premises were compensated by private insurance and liquidated their stocks as spent hens (the first scenario). Other farms that had no insurance and, therefore, received no compensation preferred to burn and bury the dead birds and sick birds by themselves (the second scenario). The large losses in the case farms under the first scenario show that insurance policies are not sufficient to cover long-term losses due to egg reduction and interrupting the production cycle. The heads of the two case farms were well educated and followed the suggestions from local veterinarians to eliminate all susceptible birds to prevent further spread that could cause even more impact on the farms in the area.

The disease caused important economic losses to affected farms averaging about 30,000 USD per farm. Most of this loss came from the opportunity cost due to removed birds, which led to egg reduction and interrupted the production cycle. This type of cost is usually neglected and is hidden from most farmers. The economic loss was communicated to farmers and local officers, leading to increased awareness about MD and the importance of vaccinating. Moreover, local authorities decided to implement intensive surveillance programs for the disease in the field.

Recommendations to tackle the outbreak were delivered to all stakeholders during the investigation, including:

- Farmers should communicate with DOCs and pullets supply companies about insurance before purchasing the DOCs or pullets
- Farmers should use a single and reliable source of birds with certificates of Marek vaccination and Marek-free birds
- If the history of vaccination is unclear, farmers should routinely send some samples to test for MD before introducing new replacement DOCs or pullets into farms and implement appropriate quarantine facilities and management, and
- Local officers, in collaboration with officers at the central level, should provide technical assistance to the companies associated with the disease to prevent other outbreaks.

## Conclusion

This study provides critical information on the value chain of layers, which can be used to effectively support MD outbreak investigations and estimate the economic impact. It provides a better understanding of the layer and egg businesses in the area, including stakeholders and their interactions, behavior, and risk practices. Moreover, the value chain information was used to guide the development of questionnaires for outbreak investigations, including the source of DOCs and pullets, risk practices, and economic figures.

From the outbreak investigation, potential risk factors for the occurrence of MD outbreaks were identified, namely, large farm size and purchasing birds from an inexperienced company that did not vaccinate against MD. Finally, the estimation of economic losses was very important in influencing the behavior of all stakeholders. Therefore, the recommendations were well accepted, and additional disease control and prevention measures were adopted.

This study has several limitations due to unavoidable circumstances. The scarcity of the data due to a limited number of case farms could have caused a non-significant effect on the potential risk factors. Farmers often do not have a good recording system of sources of chickens, egg production, number of sick and dead chickens, clinical signs, and economic parameters, which can lead to recall bias. In addition, the suspected case definition may have low sensitivity, which could cause a high number of false negatives, meaning that some positive farms were not confirmed and were not considered as case farms.

## Authors’ Contributions

TD, KC, TP, DT, and WP: Initiated the idea for the study, including the topic, the objective, and the methodology of the study. TD, KC, TP, WK, AT, DT, and WP: Collected field data and organized the database. TD and DT: Conducted the statistical and economic analysis. TD: Drafted the manuscript. TD, TS, TR, DT, and WP: Revised the manuscript. All authors have read and approved the final manuscript.
